# Ubiquitination in the ERAD Process

**DOI:** 10.3390/ijms21155369

**Published:** 2020-07-28

**Authors:** Anna Lopata, Andreas Kniss, Frank Löhr, Vladimir V. Rogov, Volker Dötsch

**Affiliations:** Institute of Biophysical Chemistry and Center for Biomolecular Magnetic Resonance, Goethe University, Max-von-Laue Str. 9, 60438 Frankfurt am Main, Germany; lopata@bpc.uni-frankfurt.de (A.L.); andikniss@aol.com (A.K.); murph@bpc.uni-frankfurt.de (F.L.); rogov@bpc.uni-frankfurt.de (V.V.R.)

**Keywords:** ERAD, ubiquitination, CUE domain, ubiquitin chain conformation

## Abstract

In this review, we focus on the ubiquitination process within the endoplasmic reticulum associated protein degradation (ERAD) pathway. Approximately one third of all synthesized proteins in a cell are channeled into the endoplasmic reticulum (ER) lumen or are incorporated into the ER membrane. Since all newly synthesized proteins enter the ER in an unfolded manner, folding must occur within the ER lumen or co-translationally, rendering misfolding events a serious threat. To prevent the accumulation of misfolded protein in the ER, proteins that fail the quality control undergo retrotranslocation into the cytosol where they proceed with ubiquitination and degradation. The wide variety of misfolded targets requires on the one hand a promiscuity of the ubiquitination process and on the other hand a fast and highly processive mechanism. We present the various ERAD components involved in the ubiquitination process including the different E2 conjugating enzymes, E3 ligases, and E4 factors. The resulting K48-linked and K11-linked ubiquitin chains do not only represent a signal for degradation by the proteasome but are also recognized by the AAA+ ATPase Cdc48 and get in the process of retrotranslocation modified by enzymes bound to Cdc48. Lastly we discuss the conformations adopted in particular by K48-linked ubiquitin chains and their importance for degradation.

## 1. Introduction

Quality-control processes are essential for every aspect of cellular function. Genetic quality-control systems ensure that the genetic information is copied with high fidelity and is efficiently repaired when damage is detected. On the protein level additional quality-control systems monitor the state of the proteome ensuring that misfolded proteins are tagged and degraded efficiently. This tight surveillance of the folding state of the proteome is critical as misfolded proteins can expose regions with high aggregation propensity which not only inactivates the affected protein but can cause the breakdown of crucial cellular functions via co-aggregation with other cellular factors [1]. The formation of prion-like fibrils is the most dramatic, as they catalyze their further growth by forcing proteins to adopt the prion-conformation. As prions are infectious and have been shown to be able to spread to uninfected cells this mechanism poses a serious threat beyond the level of a single cell but affecting tissues and the entire organism [2]. This is the most studied molecular mechanism underlying a neurodegenerative disease [3], and the mechanism could be extended to other proteins as well. As basically all proteins can transform into an amyloid state and also non-fibrils forming aggregates are dangerous, cells use several surveillance systems to monitor the integrity of their proteome [4]. These systems include a whole array of different chaperones as well as the ubiquitin-proteasome system (UPS) and the autophagy-lysosomal pathway (ALP) as the main degradation routes for proteins that do not meet quality criteria [5]. In addition to the general UPS and ALP systems, cells have developed specific systems for certain organelles that feed directly into the cellular degradation pathways but are also coupled to cell death signaling in case the stress cannot be resolved [6,7,8]. The endoplasmic reticulum (ER) plays an important role as both newly synthesized membrane proteins and soluble proteins of the secretory pathway pass through a special quality-control process [9,10] in the lumen of the ER and those that do not fold properly are removed in a process called endoplasmic reticulum associated protein degradation (ERAD) [11,12]. Approximately one third of all expressed proteins of a cell get channeled through the ER. As E3 ligases are responsible for target selection and, therefore, bind specifically to certain proteins, the vast number of different targets requires them to have a high promiscuity. To prevent aggregation of the misfolded proteins at the ER membrane the E2 enzymes involved in ERAD must be efficient and highly processive. These characteristics of the ERAD E3 and E2 enzymes in combination with the retrotranslocation of target proteins from the ER lumen/membrane to the cytosol makes the ERAD process unique within the UPS. There are several excellent reviews focusing on the entire ERAD process including the retrotranslocation mechanism [11,12,13,14,15,16]. Here, we provide a general overview of the machinery but focus on the ubiquitination process itself and discuss the various E2 and E3 enzymes involved as well as interaction with their cofactors. The result of this entire process is the attachment of a K48-linked ubiquitin chain to the target protein which functions as a specific degradation signal. We describe the various factors involved in processing of the resulting ubiquitin chain and discuss how its conformation influences its interaction with binding partners. Although the focus of this review is on the well investigated yeast system, we provide links to the mammalian counterpart as well. 

## 2. Recognition of Target Proteins

Modification of newly synthesized polypeptides in the ER lumen with asparagine-linked oligosaccharide structures serves as a marker and timer for the folding process in order to distinguish folding intermediates from terminally misfolded proteins [17,18]. The folding process is accompanied by a successive trimming of this oligosaccharide structure to finally yield a (Man)_8_GlcN(Ac)_2_ moiety. Most glycoproteins are labelled with this glycan by the time they leave the ER and after passing the quality control system. For proteins that do not pass this quality-control check-point further trimming of the oligosaccharide occurs. Processing by Htm1/Mnl1 in yeast creates a terminal α-1,6 mannose on the C-branch [19,20] of a (Man)_7_GlcN(Ac)_2_ construct. This glycan structure gets recognized by the mannose 6-phoshate receptor homology (MRH) domain of the lectin Yos9 (yeast homologue of amplified in osteosarcoma 9 protein) [21,22,23]. The mannosidase Htm1 is tightly associated with the protein-oxidoreductase Pdi1 and preferentially processes glycoproteins that display a prolonged interaction with Pdi1 based on their abnormal conformation [24,25]. The substrate selection process is further supported by Hrd3 (HMG-CoA reductase degradation 3), which is associated with Yos9, and supposed to have additional interactions with surface-exposed unstructured regions on proteins. The misfolded protein is subsequently handed over to transmembrane components of the Hrd complex. 

## 3. Retrotranslocation of the Substrate

**Figure 1:**
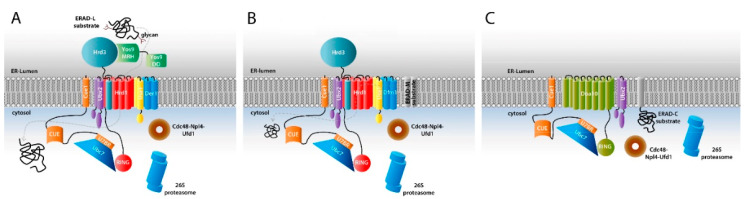
Schematic representation of major components of the ERAD machinery that are involved in the three main ERAD subtypes ERAD-L, ERAD-M, and ERAD-C. (**A**) The ERAD-L pathway. Panel shows the components of the Hrd1 complex that are required for degradation of targets localized within the ER lumen and additional important factors such as the AAA+ ATPase Cdc48 and its two associated interaction partners Npl4 and Ufd1; (**B**) The ERAD-M pathway. Panel shows the central components and associated factors required for degradation of intra-membrane substrates. This pathway does not involve Yos9 and Der1 is exchanged with Dfm1; (**C**) The ERAD-C pathway. Panel shows the components of the Doa10 complex required for degradation of proteins with misfolded cytoplasmic domains.

The recent cryo-electron microscopy studies of the Hrd complex have shown for the first time with high resolution the molecular architecture of the central component of the ERAD system and suggested a model for the retrotranslocation of misfolded proteins from the ER lumen back to the cytosol and its handover to the ubiquitination machinery [26,27]. The Hrd complex consists of the following five proteins: Hrd1, Hrd3, Der1, Usa1 and Yos9 [28,29,30] that are required for the degradation of proteins from the ER lumen (ERAD-L pathway, Figure 1A). For the degradation of ER resident membrane proteins (ERAD-M pathway, Figure 1B) only Hrd1, Hrd3, and Usa1 with the addition of the Der1 paralog, Dfm1, are required [31], while a third pathway, ERAD-C (Figure 1C) that degrades proteins with cytosolic components uses a different ER membrane embedded E3 ligase, Doa10 (degradation of alpha 10). In ERAD-L, of its five components Hrd1 is sufficient for retrotranslocation when overexpressed or *in vitro* when incorporated into proteoliposomes [32,33]. The cryo-electron microscopy structure shows that the membrane protein part of this multispanning E3 Really Interesting New Gene (RING)-finger ligase forms half of a translocation channel with the other half provided by Der1 [26]. Both half channels mark a site of a thinned membrane region that facilitates the retrotranslocation into the cellular cytosol. Recognition of the to-be-degraded substrate is achieved by the combination of the MRH domain of Yos9 that recognizes the α-1,6-mannose polysaccharide part and of a groove of the luminal domain of Hrd3 that probably binds to the polypeptide segment downstream of the glycan attachment site. Retrotranslocation is initiated by a polypeptide hairpin that is moved into the cytosol where it gets ubiquitinated followed by complete extraction of the ubiquitinated polypeptide from the ER into the cytosol by the Cdc48 adenosine triphosphatase (ATPase) and its cofactors Ufd1-Npl4 and final degradation by the proteasome (see below).

## 4. Components of the Ubiquitination Machinery

Targeting the retrotranslocated polypeptide for degradation is essential to prevent the accumulation of misfolded proteins at the membrane of the ER with potential detrimental effects. In general, ubiquitin conjugating E2 enzymes specify the ubiquitin chain linkage type and also the processivity of ubiquitination (i.e., the number of ubiquitin molecules attached to the growing chain in one round of association) [34]. Originally, only K48-linked ubiquitin chains were found to act as degradation signals [35,36]. Later it was demonstrated that also K11-linked chains, mixed chains and mono-ubiquitinated proteins [37,38] can be degraded by the proteasome. In yeast the E2 enzymes Ubc7 and Ubc6 (and to a lesser extent Ubc1) are involved in the ERAD process [39]. In mammalian cells a larger number of E2 enzymes have been identified with two homologues for each of the two yeast proteins-Ube2g1 and Ube2g2 (Ubc7) and Ube2j1 and Ube2j2 (Ubc6) [40,41,42,43,44,45]. For a summary of yeast and mammalian ERAD components see Table 1. Ubc6 and its mammalian homologues have hydrophobic C-termini that get tethered to the ER membrane via posttranslational modifications. In contrast, Ubc7 and its homologues are soluble proteins that are recruited to the membrane by interaction with Cue1, another component of the ERAD machinery (see below). Ubc7 and Ubc6 are involved in different aspects of the ERAD process. Although Ubc7 is the main E2 enzyme that cooperates with the Hrd1 E3 ligase, the central component of the Hrd complex, Ubc6 interacts with another E3 ligase complex, the Doa10 complex. Doa10 consists of 14 transmembrane helices as well as N- and C-terminal cytoplasmic domains (Figure 1C) [46]. It is not only expressed in the ER membrane but also in the nuclear envelope. In contrast to the Hrd complex that is involved in degrading proteins in the ER lumen and in the ER membrane (ERAD-L and ERAD-M pathways), Doa10 is involved in the ubiquitination of membrane proteins with cytoplasmic domains (both ER and nuclear envelope) and soluble proteins in the cytosol (ERAD-C pathway). Recently, a third E3 ligase, the Asi complex, was identified in yeast that functions exclusively at the inner membrane of the nuclear envelope [47,48] where it cooperates with the E2 enzymes Ubc7, Ubc6 and to a lesser extent with Ubc4 [48]. In the mammalian system four major E3 ligases (Hrd1, TEB4, gp78, and carboxy-terminus of Hsc70 interacting protein (CHIP)) have been identified in the ERAD process but up to 19 additional E3 ligases have been linked to the degradation of specific ER-associated targets [14,15]. Of the four major E3 ligases Hrd1 and gp78 are homologues of yeast Hrd1 and both of them bind to the Ubc7 homologue Ube2g2 [42,49,50]. In addition to its RING domain, gp78 contains a CUE domain and a G2BR domain for recruiting Ube2g2 (see below) as well. The mammalian Doa10 homologue E3 ligase is TEB4 that was found to adopt a membrane topology similar to Doa10 [46,51] and is predicted to bind to Ube2g2, Ube2j1 and Ubxd8 E2 enzymes [52] while CHIP is a U-box E3 ligase that also acts as a co-chaperone [53]. 

In yeast, the two E3 enzymes were also shown to cooperate with Ubc6 that is implicated in priming of the ubiquitin chain build-up by attaching a single ubiquitin moiety [75]. Interestingly, it seems to be able to attach ubiquitin not only to lysine residues but also to serines and possibly to threonines [75]. This ability to modify amino acids with hydroxyl group bearing side chains was also reported for the mammalian homologue of Ubc6, Ube2j2 [76]. The purpose of this relatively promiscuous mono-ubiquitination reaction could be to have many potential sites for attaching ubiquitin chains. Once a site is marked with mono-ubiquitin Ubc7 takes over which is better suited for (K48-linked) chain elongation but less for priming. In support of this model, attachment of a non-cleavable ubiquitin monomer to a substrate resulted in fast ubiquitination mediated only by Ubc7 [75]. Interestingly, it was reported that also mammalian Hrd1 can ubiquitinate non-lysine amino acids [77]. Investigation of the ubiquitination of the ERAD-L (i.e., Hrd1 dependent) substrate NS-1 κ LC (non-secreted-1 immunoglobulin κ light chain) showed that ubiquitination could still be detected when all its lysine residues were mutated to arginine. Ubiquitination by Hrd1 was only suppressed when also all serine and threonine residues were mutated as well which also resulted in inhibition of the degradation of NS-1 κ LC [77]. Likewise, it was shown that the T-cell antigen receptor α-chain becomes ubiquitinated on its cytoplasmic tail upon failure to correctly assemble the complete T-cell receptor complex. As the cytoplasmic tail of this protein does not contain any lysine residues (RLWSS), it was proposed that serines get modified which was validated by mutational analysis [78]. 

## 5. High Processivity in ERAD

### 5.1. The Special Role of the E2 Conjugating Enzymes 

**Figure 2:**
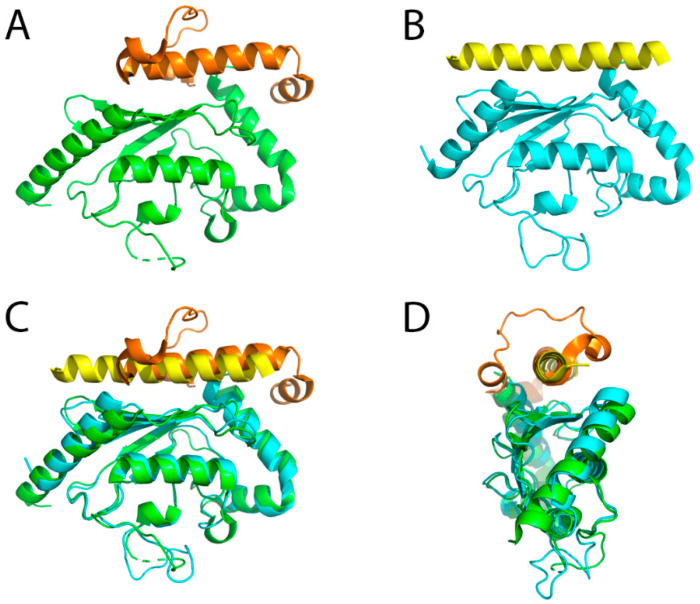
Crystal structures of E2 enzymes in complex with the U7BR/G2BR domains. (**A**) Yeast Ubc7 in complex with U7BR (PDB ID: 4JQU). The green and orange colors represent Ubc7 and U7BR, respectively; (**B**) Mammalian Ube2g2 in complex with G2BR (PDB ID: 3H8K). The cyan and yellow colors represent Ube2g2 and G2BR, respectively; (**C**) Superimposed structures of (**A**,**B**); (**D**) Superimposed structures of (**A**,**B**), rotated 90° along the vertical axis compared to (**C**). In both cases the binding domain interacts with the backside of the E2 enzyme, opposite from the catalytic cysteine.

Within the UPS system the E3 ligase is responsible for target specificity by connecting the E2 enzyme with the to-be-ubiquitinated protein. The three ERAD specific E3 ligases in yeast (including the Asi complex of the nuclear membrane), however, have to interact with a large number of diverse targets as approximately one third of all expressed proteins of a cell get channeled through the ER. As the translocation of the newly synthesized proteins into the ER lumen or into the ER membrane occurs in an unfolded form, a high risk of misfolding of a wide variety of proteins in the ER exists [79]. The required high promiscuity of the ERAD E3 ligases makes a highly efficient and processive ubiquitination reaction necessary. This is achieved by several features of the E2 conjugating enzymes. First, Ubc6 and Ubc7 have the ability to pre-assemble longer ubiquitin chains on their active site cysteine that are then transferred *en bloc* to the target protein mediated by the E3 ligases [39,80,81,82]. Second, efficient elongation of the growing ubiquitin chain is also achieved by the interaction of Ubc7 with various domains of another protein, Cue1. This protein is anchored in the ER membrane with a single transmembrane helix and has additional domains in the cytosol. One of these domains is a peptide sequence that interacts directly with Ubc7. This region is named U7BR (Ubc7 binding region) that binds to a site of the E2 enzyme opposite from the active site cysteine. The binding event recruits Ubc7 to the ER membrane, increases its local concentration in the vicinity of the ERAD E3 ligases and stabilizes Ubc7 (by preventing its degradation, see below). In addition, detailed biochemical studies have shown that binding enhances the ubiquitination reaction by an allosteric mechanism. Ubc7 bound to the isolated U7BR showed strongly enhanced ubiquitination activity, independently of the presence of E3 ligases [83]. Another study further showed that the U7BR region is the only required domain of Cue1 for ERAD if Ubc7 is tethered to the ER membrane [84]. Structure determination of a complex of Ubc7 with the U7BR region of Cue1 has revealed the molecular mechanism of interaction [85]. The U7BR domain consists of three helices that bind to the “backside” of the E2 enzyme (Figure 2A,C,D). Comparison of the apo Ubc7 structure with the structure of the Ubc7/U7BR complex did not show any major structural alterations but mainly different orientations and flexibility of the loops surrounding the active site Cys89. The observed changes in the crystal structures were further analyzed by nuclear magnetic resonance (NMR) experiments that confirmed a higher flexibility of the β4α2 loop in the complex. Consequently, charging Ubc7/U7BR with ubiquitin by an E1 enzyme is more efficient. Likewise the transfer of the thioester bound ubiquitin to another ubiquitin is accelerated and the affinity of binding to the Hrd1 RING-finger domain is increased [85]. A similar “backside” interaction is seen between the mammalian ERAD E2 enzyme Ube2g2 and the RING-finger E3 ligase gp78 [86]. Differences are, however, also evident with the Ube2g2-binding region (G2BR) of gp78 comprising only a single helix (Figure 2B–D). Structurally, the binding of the G2BR to Ube2g2 has an impact on the β4α2 and α2α3 loops as well, however, leading to a partial occlusion of the active site cysteine which also shows a different orientation of the side chain in the complex. Model building suggested that this conformation makes charging with ubiquitin by an E1 enzyme more difficult which was also confirmed experimentally. At the same time, however, binding of the G2BR to Ube2g2 enhances the affinity towards the gp78 RING-finger domain, which in this case is located in *cis* with respect to the G2BR peptide [86]. 

### 5.2. CUE Domains in the ERAD Process

In addition to the U7BR domain Cue1 contains a CUE domain. CUE domains were first identified and named after yeast Cue1 [87] for ‘coupling of ubiquitin to ER degradation’. They consist of approximately 40 amino acid residues and are semi-conserved in a range of eukaryotic proteins. In line with the origin of its name, the CUE domain containing proteins Cue1 [88], gp78 [40] and AUP1 [89] are indeed involved in the ERAD process. However, other CUE domain containing proteins such as Vps9 [90] and Tollip [91] have other functions. Originally, investigations showed that the CUE domain of Cue1 is dispensable for ubiquitination within the ERAD process. Later studies, however, demonstrated that in the presence of Hrd1 the full length Cue1 protein stimulated ubiquitination stronger than the isolated U7BR and this effect was traced to the presence of the CUE domain [92]. The same stimulation was observed when Doa10 was used as E3 ligase in these experiments showing that the CUE domain has a similar function for both the Hrd1/Ubc7 and Doa10/Ubc7-based ubiquitination reactions [92]. However, investigation of different substrate classes—soluble proteins and membrane-bound proteins—revealed that degradation of only the membrane-bound substrates was affected by either deletion of the entire CUE domain or destabilizing mutations in this domain in vivo [92].

**Figure 3:**
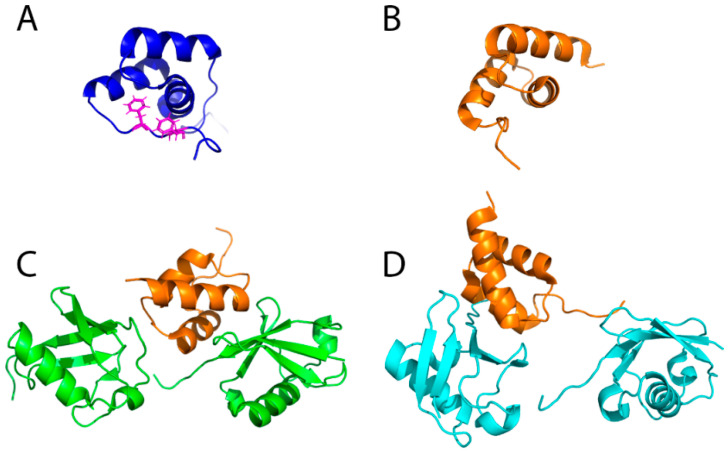
Structural comparison of CUE domains and their binding to di-ubiquitin. (**A**) NMR structure of the CUE domain of Cue1 (PDB ID: 2MYX). Phenylalanine residues that are crucial for the stability are shown with side chains; (**B**) NMR structure of the CUE domain of gp78 (PDB ID: 2LVN); (**C**) NMR structure of the CUE domain of gp78 bound to the distal moiety in a K48-linked di-ubiquitin (PDB ID: 2LVP). Orange and green colors represent the CUE domain and di-ubiquitin, respectively; (**D**) NMR structure of the CUE domain of gp78 bound to the proximal moiety in a K48-linked di-ubiquitin (PDB ID: 2LVQ). Orange and cyan colors represent the CUE domain and di-ubiquitin, respectively. The structures involving the gp78 CUE domain and di-ubiquitin show the dynamic interaction between both molecules.

**Figure 4:**
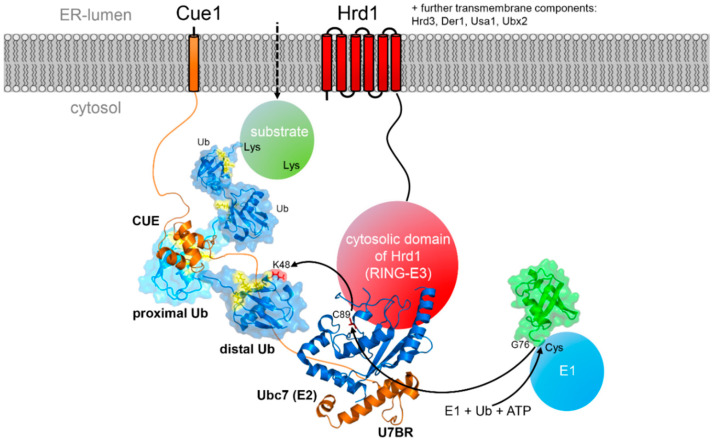
Schematic representation of the effect of the CUE domain of Cue1 on the Hrd1 dependent ubiquitination of a retrotranslocated substrate. The CUE domain preferentially binds to the proximal ubiquitin in the growing ubiquitin chain, thereby positioning Ubc7 that is also tethered to Cue1 via the U7BR domain relative to the substrate protein. Except for Hrd1 and Cue1 all other components of the Hrd1 complex are omitted for clarity.

CUE domains have been shown to be ubiquitin binding domains (UBDs) that bind to both mono-ubiquitin as well as to poly-ubiquitin chains [87,90,93,94,95]. They consist of three α-helices, similar to the ubiquitin-associated (UBA) fold. The CUE domain of yeast Cue1 differs from canonical CUE domains by requiring a C-terminal extension containing two phenylalanine residues that are crucial for stabilization of the structure (Figure 3A) [96]. All structural investigations of different CUE domains have revealed that they recognize the hydrophobic patch of ubiquitin around residues L8, I44 and V70 with dissociation constants for mono-ubiquitin in the ~10 μM range. In contrast to most CUE domains, the dissociation constant of the CUE domain of Cue1 for ubiquitin binding is high and was determined to be ~150 μM [96] most likely due to the replacement of an otherwise invariable Met-Phe-Pro triple amino acid stretch with the sequence Leu-Ala-Pro within the ubiquitin binding interface. Measuring the binding affinity of different ubiquitin chains revealed that the affinity significantly increased with increasing chain length and that K48-linked chains are preferred over K63-linked or linear chains (~90 μM for tetra K48 Ub and 110 μM for tetra K63 Ub) [96]. This result is also consistent with the observation that Ubc7 exclusively assembles K48-linked ubiquitin chains on substrates *in vivo* [80,97] and unanchored ubiquitin chains *in vitro* [92]. In general, elongation kinetics decrease with increasing chain length, an effect attributed to the growing distance between the distal end of the chain and the active center of the involved E2-E3 ligase complex which has for example been observed for the anaphase promoting complex (APC/C) [98,99]. In the presence of the CUE domain of Cue1, however, acceleration was observed with increasing chain length. The slight preference for K48-linked chains over K63-linked ones could be traced back to additional interaction with the C-terminus of the distal ubiquitin molecule within the chain which also increases the affinity to the proximal moiety (over the distal ubiquitin unit) approximately two-fold. These studies further revealed that K48 itself is not part of the interaction interface of the Cue1 CUE domain, in contrast for example to the CUE domain of Cue2 [94]. Further kinetic studies with ubiquitin chains harboring ubiquitin molecules with mutated CUE domain binding interfaces at various positions with the chain suggested a model in which the CUE domain binds preferentially to the penultimate ubiquitin in a chain [96]. The binding most likely orients Ubc7 relative to the distal end of the growing chain and thus accelerates the transfer of the next ubiquitin unit from the E2 enzyme to the acceptor lysine of the distal moiety (Figure 4).

In mammalian cells the ERAD E3 ligase gp78 contains in addition to the G2BR domain that binds to the E2 enzyme Ube2g2 also a CUE domain that recognizes the hydrophobic patch of ubiquitin similar to other CUE domains. Its interaction with di-ubiquitin is not well-defined, but dynamic and enables multiple conformations of di-ubiquitin [95], supporting growth of the ubiquitin chain and thus processivity of ubiquitination [100] (Figure 3B–D). Using NMR titrations and isothermal titration calorimetry (ITC) the binding of mono-ubiquitin and both K63- and K48-linked di-ubiquitin was measured and found to have dissociation constants in the 10–25 μM range and thus a higher affinity than the yeast Cue1 CUE domain [95]. A recent study suggested that the CUE domain of gp78 is responsible for proofreading the growing poly-ubiquitin chain to ensure K48-linkage specificity by restricting its activity for non-K48-linked chain assembly when bound to K48-linked chain [101].

### 5.3. Role of E4 Enzymes and Different Chain Linkages

The effects of the various Cue1 domains show how the concerted action of a domain that activates the E2 enzyme by “backside binding” (U7BR) and by positioning the distal end of the growing chain in an E4-like manner (CUE) facilitates ubiquitination to be highly processive. For other enzymes of the ubiquitin system it has been shown as well that processive poly-ubiquitin chain formation can be promoted by noncovalent interactions with ubiquitin. Examples are activation by “backside binding” to certain E2 enzymes [102,103] as well as by RING domains showing ubiquitin binding activity [104,105]. E4 enzymes that participate in ubiquitination reactions in yeast have been described as well and shown to be necessary for highly processive chain elongation but not in the initial steps [106]. In *in vitro* ubiquitination reactions, using the E1 enzyme Uba1, the E2 enzyme Ubc4, and the E3 ligase Ufd4 only resulted in relatively short ubiquitin chains of two to three moieties. Adding the ubiquitin binding protein Ufd2 stimulated the ubiquitination reaction, resulting in significantly longer ubiquitin chains [106]. A recent study, however, has not found an effect on ubiquitin chain elongation. Instead these investigations showed that Ufd2 adds a single ubiquitin moiety onto proximal ubiquitin molecules to form K48-ubiquitin branches [107], in particular creating branched K29/K48 ubiquitin chains on ERAD substrates. These branching reactions are necessary to target the originally K29 decorated substrates for proteasomal degradation. Ufd2 also interacts with the AAA+ ATPase Cdc48 connected to the extraction of ubiquitinated substrates from the ER [70]. The exact role of Ufd2, however, remains to be investigated.

In addition to the canonical K48-linked and branched K29/K48 chains, other chain types have also been detected on ERAD substrates. Although Ubc7 is committed to the formation of K48-linked ubiquitin chains quantitative proteomics studies have revealed that Ubc6 can be decorated with K11-linked chains in an auto-ubiquitination process and that other targets can be modified with K11-linked chains by Ubc6 as well [39]. Treating yeast with dithiothreitol (DTT) that prevents the formation of disulfide bonds in the ER or tunicamycin (inhibits N-linked glycosylation) to induce ER stress selectively increased the level of K11-linked ubiquitin chains while the level of all other linkage types remained constant. This suggested that K11-linked chains play a role via the Ubc6/Doa10 E2/E3 complex in the ERAD process [39]. Similarly, inhibiting the proteasome in mammalian cells did not only increase the abundance of ubiquitin chains with a K48-linkage, but also other chain types, in particular K11-linked ones [108].

### 5.4. Degradation of the Ubiquitination Machinery

The high and necessary promiscuity of the ERAD system for target proteins makes it not only efficient to prevent the accumulation of misfolded proteins in and at the ER but poses also great risks. This is demonstrated by overexpression experiments of Hrd1 in a Hrd3 deletion mutant strain which targets also stable ER resident proteins for degradation [29]. This observation probably also explains the growth retardation effect seen with overexpression of Hrd1 in yeast [29]. Although overexpression of Hrd1 circumvents the need for the other ERAD components by forming oligomers that are able to retrotranslocate substrate proteins [32], complex formation with Usa1 and in particular Hrd3 stabilize Hrd1 and prevent its poly-ubiquitination, extraction and degradation [109,110,111,112,113] and it has been speculated that Hrd3 regulates auto-ubiquitination of Hrd1. One effective way of down-regulating the activity of the ERAD machinery is to remove the associated E2 enzymes [32]. Ubc6 itself is an ERAD target and its degradation requires both the membrane-bound tail domain as well as the catalytic cysteine [114]. Ubiquitination of Ubc6 depends on its own catalytic function and on the activity of Ubc7, in a Doa10-dependent manner [115], consistent with the involvement of Doa10 in the degradation of ER membrane proteins with domains in the cytosol (ERAD-C pathway). Similarly, the human homologues of Ubc6, Ube2j1, and Ube2j2, were found to be targeted for proteasomal degradation and both their membrane-anchored domains and catalytic cysteines were crucial in that process [116,117]. Ubc7 also gets degraded and it was found to be down-regulated in the absence of its binding partner, Cue1 [88]. Later it was discovered that the degradation of Ubc7 involves auto-ubiquitination at the catalytic cysteine supported by Ufd4, a HECT-domain E3 ligase [80]. 

## 6. The Conformation of the Synthesized Chains

### 6.1. Binding and Modification of Ubiquitin Chains by Cdc48 and Associated Factors 

**Figure 5:**
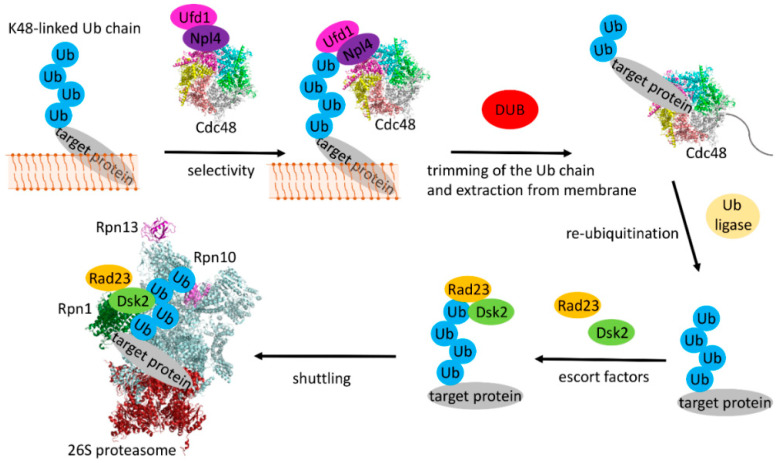
Cartoon showing the main components involved in target protein extraction and ubiquitin chain processing. For clarity, all components of the Hrd1 or Doa10 complexes are omitted. The AAA+ ATPase Cdc48 (PDB ID: 6OPC) with its two associated factors Ufd1 and Npl4 interacts with the ubiquitin chain of the target protein which leads to its extraction from the membrane. The ubiquitin chain gets processed first by trimming to an oligo-chain. After translocation through the central pore of Cdc48 the chain is again elongated, then bound to the escort factors Rad23 and Dsk2 and recruited to the proteasome for final degradation. Interaction with the proteasome is initiated by binding of the escort factors to Rpn1 and of the ubiquitin chain to Rpn10 and Rpn13 on the lid structure of the proteasome (PDB ID: 4CR2).

The ubiquitin chain attached to the substrate does not only constitute a signal for proteasomal degradation [118] but is also required to extract the ubiquitin decorated protein completely into the cytosol (in the ERAD-L pathway) [119]. There is also increasing experimental evidence that the chain is subject to extensive modifications during the extraction process (Figure 5). The final retrotranslocation of the substrate is dependent on the homohexameric AAA+ ATPase Cdc48 in yeast (or p97/valosin-containing protein (VCP) in mammalian cells) and its two cofactors Ufd1 and Npl4 [120,121]. The complex gets recruited to the ER membrane by interaction with different receptor domains located on Ubx2 (being part of both the Hrd1 and Doa10 complexes), Dfm1 and Hrd1 [31,122,123,124]. The two cofactors, Ufd1 and Npl4, contain UBDs that interact with the ubiquitinated target proteins [125,126,127] and recruit them to Cdc48 by also binding to the N-terminal domain of the AAA+ ATPase. In addition, Cdc48 interacts with several ubiquitin chain modifying enzymes and is therefore a hub for chain elongation and trimming. These modifying factors include Ufd2 [106,128,129] as well as the protein Ufd3 which does not have a chain modifying activity itself but acts as an inhibitor of Ufd2 [130]. It was also shown that the presence of Cdc48 inhibits the formation of very long ubiquitin chains and restricts the average size to three to six moieties [131] by recruiting de-ubiquitinating (DUB) enzymes such as Otu1 [132]. In yeast, the expression of an inactive Otu1 mutant (Otu1p C120S) inhibited the efficient degradation of sCPY*-DHFR and deletion of the Cdc48 binding UBX domain from the mutant DUB counter-acted this effect [123]. *In vitro* experiments with poly-ubiquitinated Hrd1 suggested that in general Cdc48 acts before Otu1-mediated trimming of the ubiquitin chains occurs [123]. These investigations also suggested that Ubx2 prevents premature de-ubiquitination before substrate extraction by competing with Otu1 for binding to Cdc48 [123]. In the mammalian system it was shown that the p97-associated DUBs Yod1 and Usp13 de-ubiquitinate substrates before they are channeled through the narrow pore of the AAA+ ATPase p97 [72]. Subsequently, they get re-ubiquitinated involving the p97 bound cofactors. Interestingly, recent studies suggest an alternative model in which Cdc48 not only unfolds the extracted substrate in an ATP-dependent manner but that trimmed ubiquitin chains pass through the central pore of the AAA+ ATPase in an unfolded state as well [123,127,132]. In this model, poly-ubiquitination is required to bind to the Ufd1/Npl4 cofactors of Cdc48. After unfolding and translocation through the pore of the AAA+ ATPase trimming of the poly-ubiquitin chain to an oligo-ubiquitin chain is necessary for substrate release, followed by translocation of this trimmed chain [132] and subsequent chain extension to create a proteasomal degradation tag (Figure 5). 

### 6.2. Interaction with the Proteasome and the Conformational Space of K48-linked Chains

**Figure 6:**
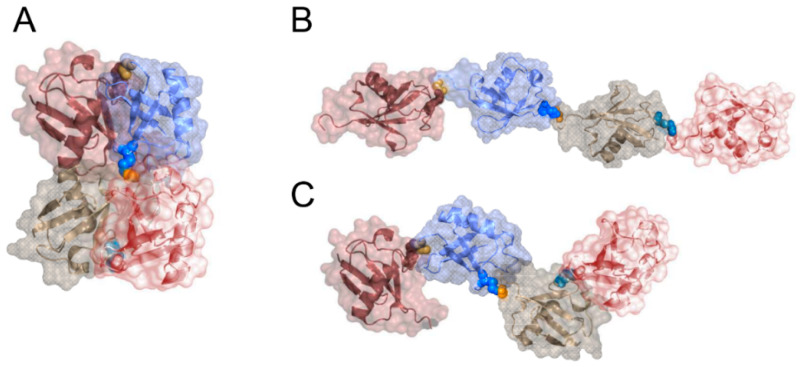
Comparison of different ubiquitin chain structures. Ubiquitin molecules are shown with both cartoon and transparent surface representation. Linking lysine residues are shown as spheres. (**A**) Crystal structure of K48-linked tetra-ubiquitin showing a compact, closed conformation with the hydrophobic patches sequestered in the complex interface (PDB ID: 2O6V); (**B**) Elongated structure of K63-linked tetra-ubiquitin (PDB ID: 3HM3); (**C**) A hypothetical elongated structure of K48-linked tetra-ubiquitin showing that due to the specific linkage type it is less elongated than the extended conformation of a K63-linked chain.

Once processed to the optimal chain length, ubiquitinated proteins are escorted to the proteasome via the soluble escort factors, Rad23p and Dsk2p [133,134] and interact with the proteasomal receptor Rpn1 [135] located on the “cap” of the proteasome (19S particle). This interaction gets further strengthened by binding of the poly-ubiquitin chain to the two ubiquitin receptors Rpn10 [136] and Rpn13 [137,138]. In all these cases the optimal length for interaction was determined to be four to six ubiquitin moieties [139,140,141]. In general, the question how different ubiquitin linkage types are recognized by interaction partners and which conformations different chain types can adopt are of central importance for understanding how the various ubiquitination patterns govern so many different cellular processes [142,143]. It was initially found that a K48-linked tetra-ubiquitin chain is the minimal length for efficient degradation [141] and it was demonstrated that the proteasomal ubiquitin receptor Rpn13 stoichiometrically binds K48-linked di-ubiquitin [137,138]. Initial studies investigated the structure of K48-linked di-ubiquitin and tetra-ubiquitin by applying various NMR techniques [144,145] and X-ray crystallography [146]. Open and closed conformations of di-ubiquitin were identified in solution and the transition was found to be dependent on the pH. Although mobility was observed at the interdomain interface at neutral pH, the structure was mainly reported to be in a closed conformation with the hydrophobic patches sequestered at the interdomain interfaces [144,145]. This was further supported by the closed and compact conformation seen in the crystal structure of K48-linked tetra-ubiquitin that was interpreted as a degradation signal [147] (Figure 6A) and by single molecule fluorescence energy transfer experiments in combination with two color coincidence detection [148]. In contrast, in a later study di-ubiquitin was observed in an open conformation, both in solution and in the crystal phase [149]. For other linkage types modelling suggested closed conformations for K6-, K11-, and K27-linked chains and extended conformations for K29-, K33- and K63-linked ones [150]. These predictions were supported for K63-linked chains of different length by X-ray crystallography [151,152,153] (Figure 6B) and solution studies [154,155]. For K11-linked chains, different conformations were seen in crystal structures showing compact structures in which the hydrophobic patches centered around I44 are solvent exposed but adopt different orientations and are located either on the same face of the dimer [156] or pointing into different directions [157]. Yet, another conformation was identified in solution studies of K11-linked di-ubiquitin in which the hydrophobic patch of the distal moiety is involved in the inter-ubiquitin interface and the hydrophobic patch of the proximal moiety is exposed [158]. Increasing salt concentrations results in more compact conformation, while pH changes have virtually no influence. Whether tetra-ubiquitin (K48-, K11-linked) adopts a closed conformation that serves as a degradation signal also poses the question how the receptors on the proteasome as well as other interaction partners should be able to bind to this compact conformation [159]. Furthermore, results showing that mono-ubiquitination on one or multiple sites constitutes a sufficient degradation signal for the proteasome [37,160] supporting the interpretation that a closed tetra-ubiquitin conformation does not represent a degradation signal. Further underlining this hypothesis more recent studies concluded that K48-linked ubiquitin chains do not adopt a predominantly closed conformation. Using NMR techniques Cook and colleagues showed that K48-linked di-ubiquitin attached to the active site of the E2 enzyme Ube2k adopts an extended conformation [161]. A mix of closed and extended conformations was also seen in crystal structures of tetra-ubiquitin [162]. Kniss and colleagues studied the entire conformational space of K48-linked di-ubiquitin and tetra-ubiquitin with pulsed electron paramagnetic resonance (EPR) and found high flexibility in both structures [163] suggesting that K48-linked ubiquitin chains adopt a wide range of conformations including completely open and closed ones (Figure 6C). Interaction studies with the CUE domain of Cue1 revealed that this conformational space gets narrower by a conformation-selection process. In contrast, interaction with an inactive form of the K48-linkage-specific Otubain protease family member OTUB1 showed a remodeling of the conformational space with conformations adopted that are not seen in free di-ubiquitin. A recent modelling study also concluded that the structure of di-ubiquitin samples a wide conformational space, with the experimentally observed conformations accounting for only 24% of all possible structures [164]. Berg and colleagues performed coarse grained simulations on differently linked di-ubiquitin and tri-ubiquitin and applied neural network-based dimensionality reduction technique to obtain a two-dimensional representation of the conformational space. Elongation seems to affect the conformational space of K48-linked ubiquitin chain, as the linking lysine residue lies in the β-sheet interaction interface that is disrupted when an additional ubiquitin moiety is added to the chain. Similar effect for K63-linked chains were not found. For both linkage types, the population of the open conformation is increased in tri-ubiquitin compared to di-ubiquitin. In conclusion, as crystal structures represent only one snapshot of a conformational ensemble, techniques yielding a conformational space reproduce more accurately the structural variety that could exist in a cell. Therefore, we propose ubiquitin chains adopt multiple conformations in the cell. Closed conformations are also possible, however not very probable, whereas open conformations are more likely to occur.

## 7. Concluding Remarks

The ER plays a special role in the cell as the central hub for protein synthesis and folding of most secreted and membrane proteins. ER stress caused by the accumulation of misfolded proteins poses a serious threat for the cell and if this stress cannot be resolved it results in the induction of apoptosis. This unique situation makes also the ERAD process unique, requiring on the one hand highly promiscuous E3 ligases that can interact with many different targets and on the other hand an efficient and processive ubiquitination reaction. Once proteins are retrotranslocated into the cytoplasm they can no longer re-enter the ER even if they would fold properly since re-entry requires a signal peptide which gets removed in the ER. Without an efficient degradation of the retrotranslocated proteins they would start to accumulate and aggregate in the cytoplasm. Many details of this process in yeast have been elucidated in the recent years. The mammalian ERAD system – in contrast – is more complicated with more E2 and E3 enzymes involved [14,15]. Interestingly, it seems that in mammalian cells target specific E3 ligases play more important roles and sometimes even different E3 proteins are responsible for degradation of differently misfolded states of the same target. One example is the chloride channel cystic fibrosis transmembrane conductance regulator (CFTR). Mutations in this membrane protein cause cystic fibrosis and mutated CFTR is a target for several ERAD associated E3 ligases. Although RING-finger protein with membrane anchor 1 (RMA1) recognizes misfolded proteins based on N-terminal mutations, CHIP is responsible for degradation of CFTR mutants, where mutations occurs in the cytoplasmic domains or in the C-terminal second nucleotide-binding domain [45,165,166]. RMA1 has also been shown to interact with gp78 as the upstream E3 ligase and gp78 acting in an E4-like manner [167], demonstrating the complexity of interactions within the mammalian ERAD associated E3 ligases. 

Another yet not fully understood topic is how the different ubiquitin linkage types are identified by the cohorts of UBDs and how different pathways are regulated by these interactions. In general, domain–domain interactions involving ubiquitin are rather weak (low μM range), enabling the adaptation of multiple conformation within ubiquitin chains and making multi-valent interactions crucial such as between the poly-ubiquitin chain and the individual ubiquitin receptors (Rpn10, Rpn13) on the S19 particle of the proteasome. Fully understanding this level of the ubiquitin code will require the characterization of the entire conformational space available to different chain types and investigation of how this space gets modulated by interaction with binding partners.

## Figures and Tables

**Table 1:** Comparison of ERAD components from yeast and mammalian cells.

Yeast	Human Homologue	Reference
***Target recognition***		
Htm1/Mnl1	EDEM1-3	[54]
Yos9	OS-9	[55]
Pdi1	Pdi	[56]
Hrd3	Sel1L	[57]
***Retrotranslocation***		
Der1	Derlin1	[58]
Dfm1	Derlin1	[58]
Usa1	HERP	[59]
Cdc48	p97	[60]
Ufd1	Ufd1	[61]
Npl4	Npl4	[62]
Ubx2	Ubxd8	[63]
***E2 ubiquitin conjugating enzymes***		
Ubc1	Ube2k	[64]
Ubc4	Ube2d1	[65]
Ubc6	Ube2j1-2	[43]
Ubc7	Ube2g1-2	[66]
***E3 ubiquitin ligase enzymes***		
Hrd1	Hrd1	[57]
Doa10	TEB4	[51]
Asi		
	gp78	[49]
	CHIP	[67]
	RMA1	[68]
***E4 ubiquitin chain elongation factors***		
Ufd2	Ufd2	[69]
Doa1	Ufd3	[70]
Cue1		
	AUP1	[71]
***Deubiquitinase***		
Otu1	Yod1	[72]
***Escort factors***		
Rad23	Hr23a-b	[73]
Dsk2	Ubiquilin-1	[74]
